# Prevalence and transmission of hepatitis E virus in domestic swine populations in different European countries

**DOI:** 10.1186/1756-0500-5-190

**Published:** 2012-04-25

**Authors:** Alessandra Berto, Jantien A Backer, Joao R Mesquita, Maria SJ Nascimento, Malcolm Banks, Francesca Martelli, Fabio Ostanello, Giorgia Angeloni, Ilaria Di Bartolo, Franco M Ruggeri, Petra Vasickova, Marta Diez-Valcarce, Marta Hernandez, David Rodriguez-Lazaro, Wim HM van der Poel

**Affiliations:** 1 Central Veterinary Institute of Wageningen Univerisity and Research Centre, Lelystad, The Netherlands; 2 Animal Health and Veterinary Laboratories Agency, Adllestone, Surrey, United Kingdom; 3 Agrarian Superior School of the Polytechnic Institute of Viseu, Department of Animal Science, Rural Engineering and Veterinary Science, Viseu, Portugal; 4 Faculty of Pharmacy of Porto University, Department of Biological Sciences, Porto, Portugal; 5 Department of Veterinary Medical Sciences, University of Bologna, Ozzano Emilia, Bologna, Italy; 6 Department of Veterinary Public Health & Food Safety, Istituto Superiore di Sanita’, Rome, Italy; 7 Instituto Tecnológico Agrario de Castilla y León (ITACyL), Junta de Castilla y León, Valladolid, Spain; 8 Veterinary Research Institute, Brno, Czech Republic; 9 National Centre for Zoonoses research, University of Liverpool, Liverpool, United Kingdom

**Keywords:** Hepatitis E virus, Foodborne disease, Pork, Foodchain, PCR, Modeling, Prevalence, European countries

## Abstract

**Background:**

Hepatitis E virus (HEV) genotype 3 and 4 can cause liver disease in human and has its main reservoir in pigs. HEV investigations in pigs worldwide have been performed but there is still a lack of information on the infection dynamics in pig populations.

**Findings:**

The HEV transmission dynamics in commercial pig farms in six different European countries was studied. The data collected show prevalence in weaners ranging from 8% to 30%. The average HEV prevalence in growers was between 20% and 44%. The fatteners prevalence ranged between 8% and 73%. Sows prevalence was similar in all countries. Boar faeces were tested for HEV only in Spain and Czech Republic, and the prevalence was 4.3% and 3.5% respectively. The collected data sets were analyzed using a recently developed model to estimate the transmission dynamics of HEV in the different countries confirming that HEV is endemic in pig farms.

**Conclusions:**

This study has been performed using similar detection methods (real time RT-PCR) for all samples and the same model (SIR model) to analyse the data. Furthermore, it describes HEV prevalence and within-herd transmission dynamics in European Countries (EU): Czech Republic, Italy, Portugal, Spain, The Netherlands and United Kingdom, confirming that HEV is circulating in pig farms from weaners to fatteners and that the reproductive number mathematical defined as R_0_ is in the same range for all countries studied.

## Findings

### Background

Hepatitis E virus (HEV) is a hepatotropic virus, causative agent of hepatitis E that has clinical and morphological characteristics of acute viral hepatitis [1,2]. In humans, the infection may vary in severity from inapparent to fulminant. The mortality is between 1% and 4%, and in pregnant women this can reach 25% [1].

Genotypes 1 and 2 appear to be mainly anthroponotic whereas genotypes 3 and 4 can be also zoonotic [3,4].

In resource-limited countries, HEV infection is endemic and spreads mainly through contamination of water supplies. Autochthonous cases have been reported in the USA, Europe, industrialized countries of the Asia–Pacific area and South America. Since the first description of a swine HEV strain in 1997 [5], swine HEV has been detected all over the world and in several animal species (e.g. wild boar, mongoose and deer). In developed regions, human and swine strains show sympatric distribution [6].

Genotype 3 has been identified in humans and animals in developed countries in almost all continents.

Higher HEV seroprevalence is detected in slaughterhouse workers and veterinarians [7,8], and it is evaluated that one third of the worldwide population has been in contact with the virus since HEV antibodies have been detected in serum [9,10].

In 2008 Di Bartolo et al. [11] investigated the prevalence of swine HEV in 274 pigs from six different swine farms of Northern Italy. Viral RNA was tested in faeces and HEV RNA was detected in 42% of the samples. All farms tested positive for HEV, with a prevalence ranging between 12.8% and 72.5%. All age groups tested HEV-RNA positive, although infection was more prevalent in weaners than in the older fatteners (42.2% vs. 27.0%).

Fernandez-Barredo et al. [12] et al. in 2006, tested 146 faecal samples of pigs from 21 farms. HEV RNA was detected in faecal samples from 34 pigs (23.29%). Pigs in the first month of feeding (60%) and weaners (41.7%) presented higher HEV prevalence.

De Deus *et al.*[13] conducted a prospective study, where 19 sows and 45 piglets were tested for antibodies to HEV. HEV IgG and IgM antibody was detected in 76.9% and 15.4% of sows, respectively. HEV RNA was also detected in serum at all analysed ages with the highest prevalence at 15 weeks of age. HEV was detected in faeces and lymph nodes for the first time at 9 weeks of age and peaked at 12 and 15 weeks of age. This peak coincided with the occurrence of hepatitis as well as with HEV detection in bile, liver, mesenteric lymph nodes and faeces, and with highest IgG and IgM OD values at 15 weeks.

Few HEV transmission dynamics studies have been performed so far. The common aim of those studies was evaluating the R_0_ that represents the number of infections that one infectious animal can cause in a fully susceptible population [14][15]. Backer et al. estimated transmission parameters to explain the prevalence pattern in pigs of different age groups. Briefly, the model describes how soon after exposure a susceptible animal can be infected (expressed by the transmission rate parameter) and how long an infectious animal excretes virus (expressed by the average infectious period).

Satou et al. [15] tried to clarify the mechanisms of transmission within farms in order to facilitate an understanding of the age-specific patterns of infection, especially just prior to slaughter.

Many HEV prevalence studies have been performed [12,16] but none of them compared HEV prevalence in different EU countries as well as in different age groups.

Hence, the aim of the present study was to evaluate HEV prevalence and HEV transmission rates in different pig age groups in different countries. For this work, results from pig samples obtained from farms in Czech Republic, Italy, Portugal, Spain, The Netherlands and United Kingdom were used. For comparison of HEV transmission rates and HEV infectious periods, the model developed by Backer *et al.* was used.

## Methods

Consensus from all farm owners was obtained previous the sample collection.

All the faeces collection was performed in conformity with standard guides, since that only faces were collected in the floor of the pigs pen and the animal were no touched at all an ethical consensus was not requested and necessary for this study.

### Samplings

The UK data sets (UK2007 and UK2008) consisted of 10 herds sampled by age class: weaners (6–9 weeks of age), growers (10–12 weeks of age), fatteners (13–22 weeks of age) and sows. Pig stool samples were collected from 10 different pig farms in 2007 and 10 pig farms in 2008. Five stool samples were obtained from each age group.

In the Portugal data set, each herd was tested at entering (weaning age of 3 weeks), growing (7 weeks) and at departure (slaughtering age of 21 weeks). A total of 200 pig stool samples were collected from 5 industrial pig farms (40 samples per farm) between December 2010 and February 2011. From each farm a total of 10 stool samples were obtained from each age group.

The data sets of Italy and The Netherlands comprised test results of one fattening group (21 weeks) of one single farm, whereas the data set obtained from Spain comprised of one group of sows in one single farm, where 144 faeces were tested for HEV RNA.

Ten pig farms were selected in Czech Republic, and a total of 200 pigs of different age groups: weaners, growers, fatteners, sows and boar where faeces were tested for HEV.

In all farms, samples of a minimum of 1 g of faeces were collected aseptically in a sterile plastic bag and maintained at 4°C (max. 24 h) or frozen at −20°C until processing.

#### RNA extraction and RT-PCR procedures

##### UK 2007, 2008 and Italy

RNA extraction and PCR is detailed by McCreary *et al.* 2008 [16]. Briefly 0.·2 g of faeces were suspended in 1.·8 ml phosphate-buffered saline, and 140 μl of the supernatant was used to extract RNA, using the QIAamp Viral RNA mini kit (Qiagen) according to the manufacturer’s instructions. The first round of the PCR used 2 μl of RNA. The reaction conditions were 96°C for five minutes, then 35 cycles of 96°C for five seconds, 55°C for five seconds and 75°C for 30 sec, followed by 72°C for one minute. A second round was carried out with a nested PCR, using a fast cycling PCR (Qiagen). These primers of the ORF-2 region are 3158 N (forward): 5′ GTT(A)ATGCTT(C)TGCATA(T)CATGGCT-3′ and 3159 N (reverse): 5′-AGCCGACGAAATCAATTCTCTC-3′ [17]. The products of the amplification process were electrophoresed, and visualised with UV light. For confirmation, the amplicons were sequenced, and the sequences obtained were assembled by using SEQMAN or DNAStar.

In Italy, RNA was processed by a reverse transcription (RT)-nested-PCR using protocol by Huang *et al.*, 2002 [17] with SuperScript One-Step RT-PCR with Platinum Taq (Invitrogen; Carlsbad, CA, USA) kit, as described in Di Bartolo et al. [18].

### The Netherlands, Portugal, Spain and Czech Republic

Two hundred and fifty mg of soft faecal contents was suspended in 2.25 ml of gentamycin-containing PBS solution and centrifuged at 3000 *g* for 15 min. Nucleic acid was extracted from 140 μl of the supernatant using the QIAamp® viral RNA mini kit (QIAGEN), according to manufacturer’s instructions.

Jothikumar’s primers and probes were used and they were designed on a multiple sequence alignment of HEV genome sequences in the ORF3 region available in GenBank [19]. Real time RT-PCR was performed using RNA Ultrasense™ One-Step Quantitative RT-PCR System (Invitrogen) and primers and probe: JHEV-F (5′- GGT GGT TTC TGG GGT GAC -3′); JVHEV-R (5′- AGG GGT TGG TTG GAT GAA -3′); JHEV-P (Taqman probe) (5′-FAM- TGA TTC TCA GCC CTT CGC –BHQ1-3′) [19]. Ten μl of RNA were added to a mix containing buffer RNA Ultrasense reaction mix (5x), ROX reference dye (50x) and RNA Ultrasense enzyme mix.

The real time RT-PCR was carried out at 50°C for 15 min, 95°C for 2 min, and 45 cycles at 95°C for 10 s, 55°C for 20 s and 72°C for 15 s.

The sensitivity of the set primers used for the HEV detection between all countries was comparable; positive (RNA of HEV positive liver) and negative (water) controls were used during the RNA extraction and during the PCRs and they worked as expected. Primers sensitivity of Huang *et al.* was 3.2 PID _50_ while Jotikimuar *et al.* sensitivity was 1.2 PDI _50_ but usually HEV in pig faeces is detectable above this values.

#### HEV transmission modelling

The model used to describe HEV transmission in a pig herd is an age-structured SIR model (Backer et al.[20]). Each age group was subdivided in three distinct compartments consisting of pigs that are susceptible (S), infectious (I) or recovered (R) [21]. For the analyses, it was assumed that each susceptible animal can be infected by an infectious animal in its own group or any other group with the same probability. The sample sizes in each data set were assumed to represent 5% of the total group size.

The transmission dynamics are characterized by the average infectious period μ and the transmission rate parameter β that signifies the number of infections one infectious animal can cause per time unit. The product of these two parameters is the reproductive number R_0_ = β μ that expresses the number of infections one infectious animal can cause during its entire infectious period in a fully susceptible population. When the reproductive number is larger than unity, R_0_ > 1, an outbreak can grow exponentially. Otherwise, when R_0_ < 1 the outbreak will die out. Our model assumes the HEV transmission to be in endemic equilibrium, i.e. the disease can sustain itself in the regenerating pig population. For this reason, we have omitted the herds with few or only negative results, as for these endemic equilibrium could not be justified.

The UK data sets (UK2007 and UK2008) consisted of herds subdivided into three groups: weaners (6–9 weeks of age), growers (10–12 weeks of age) and fatteners (13–26 weeks of age). Animals entering the weaning group were assumed to be uninfected [20]. In the Portugal data set, the herds were assumed to consist of one group that was tested at entering (weaning age of 3 weeks) and at departure (slaughtering age of 21 weeks) [20]. The test results of the growers (age of 7 weeks) were used as proxy for the infection pressure in the entire herd [20]. The data sets of Italy and The Netherlands comprised test results of just one fattening group. For this reason, we could not estimate the transmission rate parameter and the average infectious period separately, but only their product, the reproductive number [20]. For both data sets the total residence time is assumed to be 20 weeks from weaning to slaughtering age [20]. Data sets of Spain and Czech Republic were almost completely negative. For this reason, we could not estimate the reproductive number for these sets.

## Results

**Figure 1:**
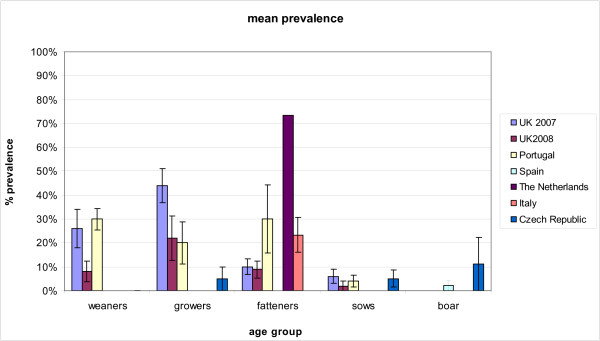
**Mean HEV prevalence in six different EU countries. **HEV prevalence plotted for six countries and 5 pig age groups. Error bars denote the standard error of the mean.

HEV prevalence in different age groups in the UK (2007, 10 farms and 2008, 10 farms), in Portugal (2011, 5 farms), Italy (2010, 3 farms), The Netherlands (2011, 1 farm), Czech Republic (2010, 10 farms), Spain (one farm between 2010 and 2011) are depicted in Figure 1. Briefly prevalence of weaners, grower’s fatteners and sows in UK 2007 was 26%, 44%, 10% and 6% respectively. Prevalence of prevalence of weaners, growers, fatteners and sows in UK 2008 was 8%, 22%, 8.8%and 2%. Prevalence of weaners, growers, fatteners and sows in Portugal was 30%, 20%, 30% and 4% respectively. Prevalence of fatteners in Italy was 23%. Prevalence of fatteners in The Netherlands was 73% meaning that 44 out of 60 pigs were shearing virus in the faeces on the day of the sample collection. The data set is similar between the age groups and prevalence match with other studies. The prevalence in The Netherlands was relatively higher in the fattening groups compared to the other European fattening groups.One hundred and forty-four faecal samples from sows collected in Spain and tested by real time RT-PCR were found to be HEV negative, while 4.3% of the boars (1 positive out of 23) were positive. In none of the weaners and fatteners tested in the CzechRepublic, HEV RNA was detected. Only one grower out of 32 (3.1%), 5 sows out of 103 (5%) and 1 boar (3.5%) out of 28 tested HEV positive by real time RT-PCR.

Table 1 shows the transmission rate parameter β, average infectious period μ and reproductive number *R*_0_ of UK 2007 and 2008 and Portugal and the reproductive number *R*_0_ for Italy and The Netherlands. The data set from Spain and Czech Republic could not be used in this study since all or almost all animal tested were HEV negative and we could not apply the model to those data.

**Table 1:** Estimated transmission rate parameters

**dataset**	**transmission rate parameter β (day**^**-1**^**)**		**average infectious period μ (days)**		**reproductive number *R***_**0**_	
UK 2007 (10 herds)	0.11	(0.070 – 0.17)	43	(33 – 59)	4.7	(3.6 – 6.4)
UK 2008 (8 herds)	0.071	(0.041 – 0.13)	43	(29 – 73)	3.1	(2.5 – 4.1)
Portugal (6 herds)	0.037	(0.0035 – 0.16)	101	(70–403)	3.7	(1.2 – 14)
Italy (3 herds)	-		-		2.0	(1.4 – 3.6)
Netherlands (1 herd)	-		-		8.4	(5.3 – 15)
Spain	-		-		-	-
Czech Republic	-		-		-	-

Median maximum likelihood estimates and 5% – 95% credible interval between brackets.

## Discussion

The HEV transmission dynamics in commercial pig farms in six different European countries (UK, Portugal, Italy, The Netherlands, Spain and Czech Republic) was studied.

The data collected show prevalence in weaners ranging from 8% to 30%. The average HEV prevalence in growers was between 20% and 44%. The fatteners prevalence ranged between 8% and 73%. Sows prevalence was similar in all countries. Boar faeces were tested for HEV only in Spain and Czech Republic, and the prevalence was 4.3% and 3.5% respectively.

Overall, Figure 1 describes HEV prevalence comparing Czech Republic, Italy, Portugal, Spain, The Netherlands and UK 2007, 2008. The data set is similar between the age groups and prevalence matches with other studies [12,16]. The prevalence in the fattening groups in Italy and The Netherlands was relatively higher compared to other European fattening groups [21].

These data are similar to previously published Italian [11] and Spanish [12] data, confirming that HEV prevalence during time is constant and HEV is circulating in all farms in all age groups, from weaners to fatteners and that pigs close to the slaughter age can still be HEV infected.

The collected data sets were analyzed using a recently developed model to estimate the transmission dynamics of HEV in the different countries [20].

Satou *et al.* in 2007 [15] studied HEV transmission in 6 different Japanese provinces and found the reproductive number in the order of 4.02 – 5.17, which agrees with our estimated reproductive numbers ranging from 2.0 to 8.4. The study by Satou et al. [15] was the first report on HEV transmission estimated from field data. Bouwknegt *et al.* in 2008 performed the first HEV transmission dynamics study in an animal experiment [22]. In this study, the R_0_ was found to be 8.8 and 32 in two separate experiments, much higher than 1.0 indicating that swine could be assumed to be a true reservoir of HEV. The R_0_ values calculated by us are lower than the R_0_ values calculated by Bouwknegt et al. [22]. This is because the infectious periods are comparable, but the transmission rate parameters for the experimental and field situation are different.

The average infectious period μ in UK 2007 data was for instance estimated to be 43 (33 – 59) days, whereas Bouwknegt *et al.*[22] estimated average infectious periods of 49 (17–141) days and 13 (11 – 17) days.

The transmission rate parameter in our study was 0.11 (0.070 – 0.17) day^-1^ for UK 2007, meaning that one infectious animal infects another animal every 10 days. The transmission rate parameters were 0.071 (0.041-0.13) day^-1^ for UK 2008 and 0.037 (0.0035-0.16) day^-1^ for Portugal 2011. In the animal experiments, Bouwknegt et al. [22] estimated a higher rate of transmission, i.e. 0.66 (95% CI: 0.32–1.35) day^-1^. The difference can be explained by the closer proximity of animals in an experimental setting compared to a farm situation and by the fact that contact animals in a transmission experiment encounter only animals that are in the early and possibly more infectious stages of virus shedding.

The transmission rate parameters for the other EU countries could not be estimated because either only one age group was tested or the majority of the animals were negative and the model was not applicable.

This study gave a genuine contribution to better understand HEV prevalence in six different European countries by a mathematical model.

We would like to highlight that HEV is highly circulating in many pig farms in Europe and can be present in fattening pigs, where usually this age group is the one arriving to the table. In industrialized regions, although the incidence of clinical hepatitis E in humans is low, the seroprevalence is relatively high [22], indicating a high proportion of subclinical disease and/or underdiagnosis [8]. It is likely that a small proportion of this exposure to HEV results from travel to endemic regions, or migration from endemic regions [23], this still leaves a substantial level of exposure to HEV that appears to have an indigenous source.

HEV positive fatteners were found in all European countries studied. This may pose an important risk for public health especially in those countries where pork products are eaten undercooked or raw.

## Competing interests

There are no competing interests relating to any of the authors.

## Author’s contribution

*First author biographical*: Ms Alessandra Berto is a final year PhD student on the VITAL project and is based at the AHVLA Weybridge, UK and the Wageningen University Research Institute in The Netherlands. She tested and collected some of the data and she helped JB to set up the model. JA Backer was the modeler. JR Mesquita MSJ Nascimento provided the Portuguese samples. M. Banks, F. Martelli provided the Uk data set. F. Ostanello,G. Angeloni, I. Di Bartolo, F. M. Ruggeri : provided the Italian data set. P. Vasickova provided the Cechz republic data set. Diez-Valcarce, M. Hernandez, D. Rodriguez-Lazaro: provided the Spainish data set. W.H.M. van der Poel was involved in drafting the manuscript or revising it critically for important intellectual content and gave final approval of the version to be published. This manuscript has been read and approved by all authors.

## References

[B1] Purcell RH Emerson SU Hepatitis E: an emerging awareness of an old disease J Hepatol 2008 48 3 494 503 10.1016/j.jhep.2007.12.008 18192058

[B2] Meng XJ Recent advances in Hepatitis E virus J Viral Hepat 2010 17 3 153 161 10.1111/j.1365-2893.2009.01257.x 20040046

[B3] Wei H Zhang JQ Lu HQ Meng JH Lu XX Xie W Construction and screening of hepatitis E virus-specific phage antibody combinatorial library Xi Bao Yu Fen Zi Mian Yi Xue Za Zhi 2003 19 5 473 475, 485 15169661

[B4] Schlauder GG Mushahwar IK Genetic heterogeneity of hepatitis E virus J Med Virol 2001 65 2 282 292 10.1002/jmv.2031 11536234

[B5] Meng XJ Purcell RH Halbur PG Lehman JR Webb DM Tsareva TS Haynes JS Thacker BJ Emerson SU A novel virus in swine is closely related to the human hepatitis E virus Proc Natl Acad Sci U S A 1997 94 18 9860 9865 10.1073/pnas.94.18.9860 9275216 PMC23282

[B6] Banks M Heath GS Grierson SS King DP Gresham A Girones R Widen F Harrison TJ Evidence for the presence of hepatitis E virus in pigs in the United Kingdom Vet Rec 2004 154 8 223 227 10.1136/vr.154.8.223 15005446

[B7] Vulcano A Angelucci M Candelori E Martini V Patti AM Mancini C Santi AL Calvani A Casagni L Lamberti A HEV prevalence in the general population and among workers at zoonotic risk in Latium Region Ann Ig 2007 19 3 181 186 17658105

[B8] Meng XJ Wiseman B Elvinger F Guenette DK Toth TE Engle RE Emerson SU Purcell RH Prevalence of antibodies to hepatitis E virus in veterinarians working with swine and in normal blood donors in the United States and other countries J Clin Microbiol 2002 40 1 117 122 10.1128/JCM.40.1.117-122.2002 11773103 PMC120098

[B9] Yazaki Y Mizuo H Takahashi M Nishizawa T Sasaki N Gotanda Y Okamoto H Sporadic acute or fulminant hepatitis E in Hokkaido, Japan, may be food-borne, as suggested by the presence of hepatitis E virus in pig liver as food J Gen Virol 2003 84 Pt 9 2351 2357 12917455 10.1099/vir.0.19242-0

[B10] Colson P Borentain P Queyriaux B Kaba M Moal V Gallian P Heyries L Raoult D Gerolami R Pig liver sausage as a source of hepatitis E virus transmission to humans J Infect Dis 2010 202 6 825 834 10.1086/655898 20695796

[B11] Martelli F Caprioli A Zengarini M Marata A Fiegna C Di Bartolo I Ruggeri FM Delogu M Ostanello F Detection of hepatitis E virus (HEV) in a demographic managed wild boar (Sus scrofa scrofa) population in Italy Vet Microbiol 2008 126 1–3 74 81 17706898 10.1016/j.vetmic.2007.07.004

[B12] Fernandez-Barredo S Galiana C Garcia A Vega S Gomez MT Perez-Gracia MT Detection of hepatitis E virus shedding in feces of pigs at different stages of production using reverse transcription-polymerase chain reaction J Vet Diagn Invest 2006 18 5 462 465 10.1177/104063870601800506 17037614

[B13] de Deus N Casas M Peralta B Nofrarias M Pina S Martin M Segales J Hepatitis E virus infection dynamics and organic distribution in naturally infected pigs in a farrow-to-finish farm Vet Microbiol 2008 132 1–2 19 28 18562132 10.1016/j.vetmic.2008.04.036

[B14] Greenhalgh D Diekmann O de Jong MC Subcritical endemic steady states in mathematical models for animal infections with incomplete immunity Math Biosci 2000 165 1 1 25 10.1016/S0025-5564(00)00012-2 10804257

[B15] Satou K Nishiura H Transmission dynamics of hepatitis E among swine: potential impact upon human infection BMC Vet Res 2007 3 9 10.1186/1746-6148-3-9 17493260 PMC1885244

[B16] McCreary C Martelli F Grierson S Ostanello F Nevel A Banks M Excretion of hepatitis E virus by pigs of different ages and its presence in slurry stores in the United Kingdom Vet Rec 2008 163 9 261 265 10.1136/vr.163.9.261 18757902

[B17] Huang FF Haqshenas G Guenette DK Halbur PG Schommer SK Pierson FW Toth TE Meng XJ Detection by reverse transcription-PCR and genetic characterization of field isolates of swine hepatitis E virus from pigs in different geographic regions of the United States J Clin Microbiol 2002 40 4 1326 1332 10.1128/JCM.40.4.1326-1332.2002 11923352 PMC140370

[B18] Di Bartolo I Ponterio E Castellini L Ostanello F Ruggeri FM Viral and antibody HEV prevalence in swine at slaughterhouse in Italy Vet Microbiol 2011 149 3–4 330 338 21216541 10.1016/j.vetmic.2010.12.007

[B19] Jothikumar N Cromeans TL Robertson BH Meng XJ Hill VR A broadly reactive one-step real-time RT-PCR assay for rapid and sensitive detection of hepatitis E virus J Virol Methods 2006 131 1 65 71 10.1016/j.jviromet.2005.07.004 16125257

[B20] Backer AB JA McCreary C Martelli F WHM van der Poel Transmission dynamics of Hepatitis E virus in pigs: estimation from field data and effect of vaccinationsubmitted to Epidemics 2012 10.1016/j.epidem.2012.02.002 22664067

[B21] Di Bartolo I Martelli F Inglese N Pourshaban M Caprioli A Ostanello F Ruggeri FM Widespread diffusion of genotype 3 hepatitis E virus among farming swine in Northern Italy Vet Microbiol 2008 132 1–2 47 55 18538512 10.1016/j.vetmic.2008.04.028

[B22] Bouwknegt M Frankena K Rutjes SA Wellenberg GJ de Roda Husman AM van der Poel WH de Jong MC Estimation of hepatitis E virus transmission among pigs due to contact-exposure Vet Res 2008 39 5 40 10.1051/vetres:2008017 18367077

[B23] Peron JM Mansuy JM Poirson H Bureau C Dupuis E Alric L Izopet J Vinel JP Hepatitis E is an autochthonous disease in industrialized countries. Analysis of 23 patients in South-West France over a 13-month period and comparison with hepatitis A Gastroenterol Clin Biol 2006 30 5 757 762 10.1016/S0399-8320(06)73310-3 16801899

